# Crystal structure of (2*E*)-*N*-methyl-2-(2-oxo-1,2-di­hydroacenaphthylen-1-ylidene)hydrazinecarbo­thioamide

**DOI:** 10.1107/S1600536814023216

**Published:** 2014-10-24

**Authors:** G. Vimala, J. Govindaraj, J. Haribabu, R. Karvembu, A. SubbiahPandi

**Affiliations:** a Department of Physics, Presidency College (Autonomous), Chennai 600 005, India; b Department of Physics, Pachaiyappa’s College for Men, Kancheepuram 631 501, India; c Department of Chemistry, National Institute of Technology, Trichy 620 015, India

**Keywords:** crystal structure, ace­naphthyl­ene, hydrazinecarbo­thio­amide, thio­semicarbazones, hydrogen bonding, C—H⋯π inter­actions

## Abstract

In the title compound, C_14_H_11_N_3_OS, the ace­naphthyl­ene ring system and hydrazinecarbo­thio­amide unit (=N—NH—C=S—NH–) are essentially coplanar [with maximum deviations from their mean planes of −0.009 (2) and 0.033 (2) Å, respectively], and make a dihedral angle of 1.59 (9)°. The mol­ecular conformation is stabilized by two weak intra­molecular hydrogen bonds (N—H⋯O and N—H⋯N), which generate *S*(6) and *S*(5) ring motifs. In the crystal, mol­ecules are linked by N—H⋯S hydrogen bonds, forming chains along [010]. The chains are linked *via* pairs of C—H⋯O hydrogen bonds, enclosing *R* ^2^_2_(10) ring motifs, and C—H⋯π inter­actions, forming a three-dimensional framework. The absolute structure of the title compound was determined by resonant scattering.

## Abstract 2

In the title compound, the acenapthylene ring system and the hydrazinecarbo­thio­amide unit (=N—NH—C=S—NH–) are essentially coplanar, making a dihedral angle of 1.59 (9)°. The mol­ecular conformation is stabilized by two weak intra­molecular hydrogen bonds (N—H⋯O and N—H⋯N), which generate *S*(6) and *S*(5) ring motifs.

## Chemical context   

The design and synthesis of thio­semicarbazones are of considerable inter­est because of their versatile chemistry and various biological activities, such as anti­tumor, anti­bacterial, anti­viral, anti­amoebic and anti­malarial (Kelly *et al.*, 1996 ▶). They comprise an intriguing class of chelating mol­ecules, which possess a wide range of beneficial medicinal properties (Prabhakaran *et al.* 2008 ▶). Thio­semicarbazones are a versatile class of ligands that have been studied for their biological activity (Chellan *et al.*, 2010 ▶), their inter­esting binding motifs (Lobana *et al.*, 2009 ▶) and their use as ligands in catalysis (Xie *et al.*, 2010 ▶). In view of their biological importance, the crystal structure of the title compound has been determined and the results are presented herein.
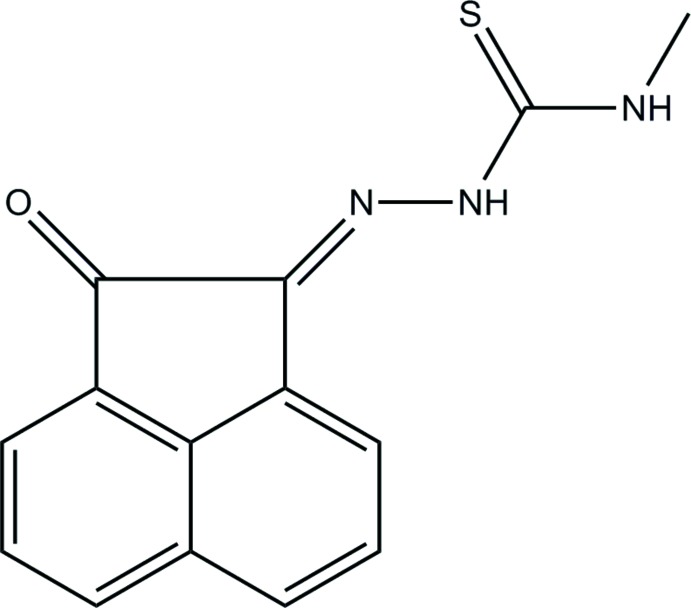


## Structural commentary   

**Figure 1:**
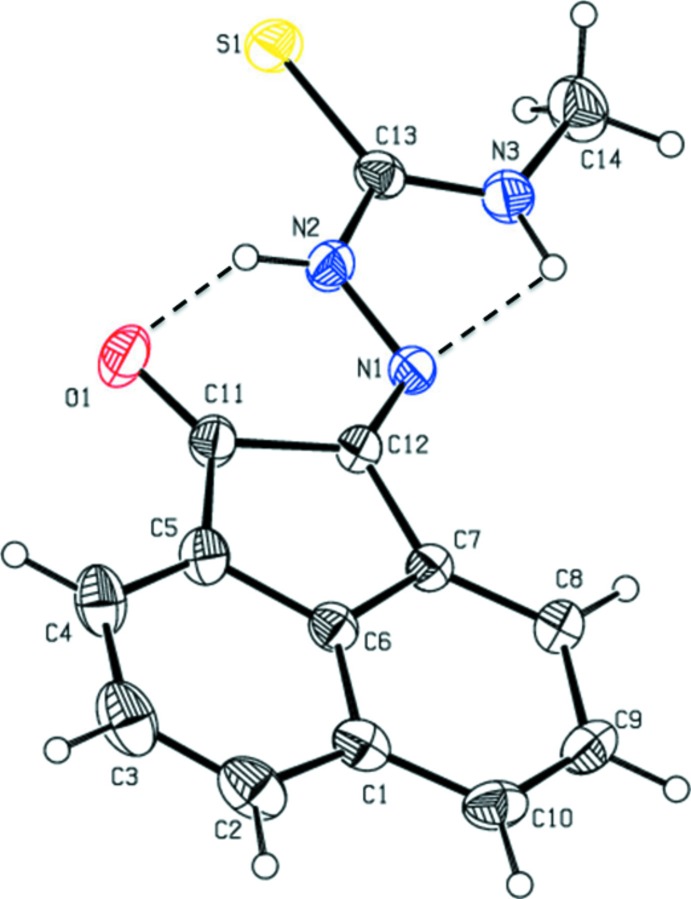
The mol­ecular structure of the title compound, with the atom labelling. Displacement ellipsoids are drawn at the 30% probability level. Hydrogen bonds are shown as dashed lines (see Table 1 ▶ for details).

The mol­ecular structure of the title compound is illustrated in Fig. 1 ▶. The atoms of both the ace­naphthyl­ene ring system and the =N—NH—C=S—NH– segment are essentially coplanar, the maximum deviations from their mean planes being −0.009 (2) and 0.033 (2) Å for atoms C12 and C14, respectively. The dihedral angle between the benzene and cyclo­pentane rings of the acenapthalene unit is 1.59 (9)°. The mol­ecular structure is stabilized by N—H⋯O and N—H⋯N hydrogen bonds, forming *S*(6) and *S*(5) ring motifs, respectively (Table 1 ▶ and Fig. 1 ▶).

## Supra­molecular features   

**Figure 2:**
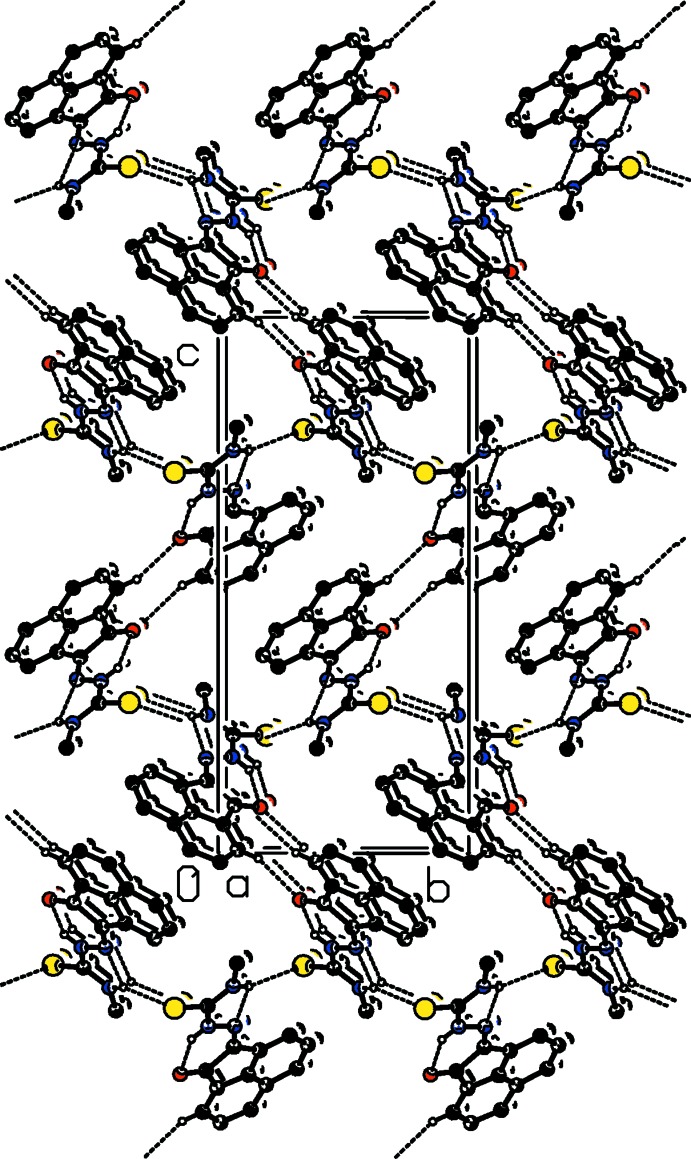
The crystal packing of the title compound viewed along the *a* axis. Hydrogen bonds are shown as dashed lines (see Table 1 ▶ for details; H atoms not involved in hydrogen bonding have been omitted for clarity).

In the crystal, mol­ecules are linked by N—H⋯S hydrogen bonds (Table 1 ▶ and Fig. 2 ▶), forming chains along [010]. The chains are linked *via* pairs of C—H⋯O hydrogen bonds, enclosing 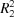
(10) ring motifs, and C—H⋯π inter­actions, forming a three-dimensional framework (Table 1 ▶ and Fig. 2 ▶).

## Database survey   

A search of the Cambridge Structural Database (Version 5.35, last update May 2014; Groom & Allen, 2014 ▶) for the substructure 2-(imino)­ace­naphthylen-1(2*H*)-one gave 13 hits, including that of the ethyl analogue of the title compound, ace­naphthyl­ene-1,2-dione 4-ethyl­thio­semicarbazone (GUR­HAD; Pascu *et al.*, 2010 ▶). The two mol­ecules differ in the dihedral angle between the mean planes of the ace­naphthyl­ene ring system and hydrazinecarbo­thio­amide unit (=N—NH—C=S—NH–) which is 1.59 (9)° in the title compound but 9.14 (6)° in the ethyl analogue (GURHAD; Pascu *et al.*, 2010 ▶). In the crystals of both compounds, mol­ecules are linked *via* N—H⋯S hydrogen bonds, forming chains along [010].

## Synthesis and crystallization   

An ethano­lic solution of *N*-methyl­hydrazinecarbo­thio­amide (0.01 mol) was added to an ethano­lic solution (50 ml) containing ace­naphthyl­ene-1,2-dione (0.01 mol). The mixture was refluxed for 2 h during which time a yellow precipitate separated out. The reaction mixture was then cooled to room temperature and the precipitate was filtered off. It was then washed with ethanol and dried under vacuum. The yield of the isolated product was 89%. Single crystals suitable for X-ray diffraction were obtained by slow evaporation of a solution of the title compound in ethanol at room temperature.

## Refinement   

Crystal data, data collection and structure refinement details are summarized in Table 2 ▶. All H atoms were fixed geom­etrically and allowed to ride on their parent atoms: N—H = 0.86 and C—H = 0.93–0.97 Å and with *U*_iso_(H) = 1.5*U*_eq_(C) for methyl H atoms and = 1.2*U*_eq_(C) for other H atoms. The absolute structure of the title compound was determined by resonant scattering, with a Flack parameter of 0.02 (8).

## Supplementary Material

Crystal structure: contains datablock(s) I, global. DOI: 10.1107/S1600536814023216/su2796sup1.cif

Structure factors: contains datablock(s) I. DOI: 10.1107/S1600536814023216/su2796Isup2.hkl

Supporting information file. DOI: 10.1107/S1600536814023216/su2796Isup3.cml

CCDC reference: 1030348

Additional supporting information:  crystallographic information; 3D view; checkCIF report

## Figures and Tables

**Table 1:** Hydrogen-bond geometry (, ) *Cg* is the centroid of ring C1/C6C10.

*D*H*A*	*D*H	H*A*	*D* *A*	*D*H*A*
N2H2O1	0.86	2.03	2.7178(19)	136
N3H3N1	0.86	2.26	2.6437(19)	107
N3H3S1^i^	0.86	2.64	3.4407(15)	156
C4H4O1^ii^	0.93	2.47	3.246(2)	141
C2H2*A* *Cg* ^iii^	0.93	2.76	3.502(2)	137

Symmetry codes: (i) 
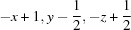
; (ii) 
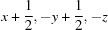
; (iii) 
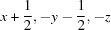
.

**Table 2:** Experimental details

Crystal data	
Chemical formula	C_14_H_11_N_3_OS
*M*_r_	269.33
Crystal system, space group	Orthorhombic, *P*2_1_2_1_2_1_
Temperature (K)	293
*a*, *b*, *c* ()	6.1110(6), 10.0547(11), 21.497(2)
*V* (^3^)	1320.8(2)
*Z*	4
Radiation type	Mo *K*
(mm^1^)	0.24
Crystal size (mm)	0.30 0.25 0.20

Data collection	
Diffractometer	Bruker *SMART* APEXII CCD
Absorption correction	Multi-scan (*SADABS*; Bruker, 2008 ▶)
*T*_min_, *T*_max_	0.932, 0.954
No. of measured, independent and observed [*I* > 2(*I*)] reflections	23135, 3941, 2929
*R*_int_	0.030
(sin /)_max_ (^1^)	0.708

Refinement	
*R*[*F* ^2^ > 2(*F* ^2^)], *wR*(*F* ^2^), *S*	0.035, 0.100, 0.99
No. of reflections	3941
No. of parameters	173
H-atom treatment	H-atom parameters constrained
_max_, _min_ (e ^3^)	0.19, 0.21
Absolute structure	Flack (1983 ▶); Friedel pairs
Absolute structure parameter	0.02(8)

Computer programs: *APEX2* and *SAINT* (Bruker, 2008 ▶), *SHELXS97* and *SHELXL97* (Sheldrick, 2008 ▶), *ORTEP-3 for Windows* (Farrugia, 2012 ▶) and *PLATON* (Spek, 2009 ▶).

## supplementary crystallographic information

### Crystal data

**Table:**

C_14_H_11_N_3_OS	*Z* = 4
*M**_r_* = 269.33	*F*(000) = 560
Orthorhombic, *P*2_1_2_1_2_1_	*D*_x_ = 1.354 Mg m^−^^3^
Hall symbol: P 2ac 2ab	Mo *K*α radiation, λ = 0.71073 Å
*a* = 6.1110 (6) Å	µ = 0.24 mm^−^^1^
*b* = 10.0547 (11) Å	*T* = 293 K
*c* = 21.497 (2) Å	Block, yellow
*V* = 1320.8 (2) Å^3^	0.30 × 0.25 × 0.20 mm

### Data collection

**Table:**

Bruker SMART APEXII CCD diffractometer	3941 independent reflections
Radiation source: fine-focus sealed tube	2929 reflections with *I* > 2σ(*I*)
Graphite monochromator	*R*_int_ = 0.030
ω and φ scans	θ_max_ = 30.2°, θ_min_ = 2.2°
Absorption correction: multi-scan (*SADABS*; Bruker, 2008)	*h* = −8→8
*T*_min_ = 0.932, *T*_max_ = 0.954	*k* = −13→14
23135 measured reflections	*l* = −29→29

### Refinement

**Table:**

Refinement on *F*^2^	Secondary atom site location: difference Fourier map
Least-squares matrix: full	Hydrogen site location: inferred from neighbouring sites
*R*[*F*^2^ > 2σ(*F*^2^)] = 0.035	H-atom parameters constrained
*wR*(*F*^2^) = 0.100	*w* = 1/[σ^2^(*F*_o_^2^) + (0.0532*P*)^2^ + 0.2048*P*] where *P* = (*F*_o_^2^ + 2*F*_c_^2^)/3
*S* = 0.99	(Δ/σ)_max_ = 0.001
3941 reflections	Δρ_max_ = 0.19 e Å^−^^3^
173 parameters	Δρ_min_ = −0.21 e Å^−^^3^
0 restraints	Absolute structure: Flack (1983); Friedel pairs
Primary atom site location: structure-invariant direct methods	Absolute structure parameter: −0.02 (8)

### Special details

**Table:**

Geometry. All e.s.d.'s (except the e.s.d. in the dihedral angle between two l.s. planes) are estimated using the full covariance matrix. The cell e.s.d.'s are taken into account individually in the estimation of e.s.d.'s in distances, angles and torsion angles; correlations between e.s.d.'s in cell parameters are only used when they are defined by crystal symmetry. An approximate (isotropic) treatment of cell e.s.d.'s is used for estimating e.s.d.'s involving l.s. planes.
Refinement. Refinement of *F*^2^ against ALL reflections. The weighted *R*-factor *wR* and goodness of fit *S* are based on *F*^2^, conventional *R*-factors *R* are based on *F*, with *F* set to zero for negative *F*^2^. The threshold expression of *F*^2^ > σ(*F*^2^) is used only for calculating *R*-factors(gt) *etc*. and is not relevant to the choice of reflections for refinement. *R*-factors based on *F*^2^ are statistically about twice as large as those based on *F*, and *R*-factors based on ALL data will be even larger.

### Fractional atomic coordinates and isotropic or equivalent isotropic displacement parameters (Å^2^)

**Table:**

	*x*	*y*	*z*	*U*_iso_*/*U*_eq_	
S1	0.25692 (8)	0.16757 (4)	0.21243 (2)	0.04960 (13)	
N1	0.7542 (2)	−0.05741 (12)	0.17414 (6)	0.0365 (3)	
C6	1.2232 (3)	−0.09719 (15)	0.07905 (7)	0.0371 (3)	
O1	0.8428 (2)	0.16301 (14)	0.08374 (6)	0.0550 (4)	
N2	0.6084 (2)	0.04380 (14)	0.17558 (7)	0.0416 (3)	
H2	0.6259	0.1109	0.1513	0.050*	
C7	1.0847 (3)	−0.14650 (15)	0.12619 (7)	0.0342 (3)	
C13	0.4342 (3)	0.04074 (16)	0.21510 (8)	0.0380 (3)	
N3	0.4208 (2)	−0.06205 (15)	0.25248 (7)	0.0445 (3)	
H3	0.5225	−0.1210	0.2506	0.053*	
C12	0.9103 (3)	−0.04864 (16)	0.13442 (7)	0.0350 (3)	
C5	1.1533 (3)	0.02565 (17)	0.05510 (8)	0.0425 (4)	
C11	0.9539 (3)	0.06370 (17)	0.08912 (8)	0.0403 (4)	
C8	1.1346 (3)	−0.26514 (17)	0.15336 (8)	0.0416 (4)	
H8	1.0445	−0.3015	0.1838	0.050*	
C9	1.3268 (3)	−0.3313 (2)	0.13404 (9)	0.0492 (4)	
H9	1.3632	−0.4114	0.1530	0.059*	
C14	0.2450 (4)	−0.0824 (2)	0.29673 (10)	0.0634 (5)	
H14A	0.2421	−0.0099	0.3258	0.095*	
H14B	0.2686	−0.1642	0.3187	0.095*	
H14C	0.1080	−0.0866	0.2750	0.095*	
C1	1.4122 (3)	−0.16161 (19)	0.05877 (8)	0.0435 (4)	
C2	1.5292 (4)	−0.0968 (2)	0.01114 (10)	0.0604 (6)	
H2A	1.6559	−0.1356	−0.0045	0.072*	
C4	1.2699 (4)	0.0860 (2)	0.00894 (9)	0.0578 (5)	
H4	1.2252	0.1668	−0.0078	0.069*	
C3	1.4596 (4)	0.0221 (2)	−0.01250 (11)	0.0671 (6)	
H3A	1.5408	0.0621	−0.0439	0.080*	
C10	1.4618 (3)	−0.2829 (2)	0.08867 (9)	0.0505 (5)	
H10	1.5867	−0.3300	0.0775	0.061*	

### Atomic displacement parameters (Å^2^)

**Table:**

	*U*^11^	*U*^22^	*U*^33^	*U*^12^	*U*^13^	*U*^23^
S1	0.0421 (2)	0.0413 (2)	0.0654 (3)	0.0080 (2)	0.0033 (2)	−0.01024 (19)
N1	0.0345 (6)	0.0331 (6)	0.0420 (7)	0.0008 (6)	0.0020 (7)	−0.0007 (5)
C6	0.0373 (9)	0.0370 (8)	0.0369 (8)	−0.0031 (7)	−0.0004 (7)	−0.0047 (6)
O1	0.0621 (8)	0.0423 (7)	0.0605 (8)	0.0123 (7)	0.0046 (7)	0.0140 (6)
N2	0.0397 (7)	0.0361 (7)	0.0488 (8)	0.0054 (6)	0.0055 (7)	0.0043 (6)
C7	0.0353 (8)	0.0338 (8)	0.0336 (7)	0.0004 (6)	−0.0001 (6)	−0.0013 (6)
C13	0.0337 (8)	0.0365 (8)	0.0438 (8)	−0.0028 (6)	0.0001 (7)	−0.0089 (7)
N3	0.0401 (8)	0.0406 (7)	0.0528 (8)	0.0015 (6)	0.0077 (7)	−0.0009 (7)
C12	0.0356 (8)	0.0326 (7)	0.0368 (7)	0.0007 (6)	−0.0014 (7)	0.0021 (6)
C5	0.0498 (10)	0.0395 (9)	0.0381 (8)	−0.0029 (8)	0.0047 (8)	−0.0009 (7)
C11	0.0436 (9)	0.0357 (8)	0.0415 (8)	−0.0005 (7)	−0.0003 (7)	0.0040 (7)
C8	0.0454 (10)	0.0386 (9)	0.0407 (9)	0.0029 (8)	0.0000 (8)	0.0019 (7)
C9	0.0523 (10)	0.0432 (9)	0.0521 (10)	0.0129 (9)	−0.0088 (8)	−0.0013 (8)
C14	0.0583 (12)	0.0610 (12)	0.0707 (13)	−0.0037 (11)	0.0226 (12)	0.0021 (9)
C1	0.0388 (9)	0.0476 (9)	0.0441 (9)	−0.0044 (8)	0.0042 (7)	−0.0129 (8)
C2	0.0513 (12)	0.0702 (14)	0.0596 (12)	−0.0090 (10)	0.0196 (10)	−0.0180 (10)
C4	0.0742 (14)	0.0496 (10)	0.0495 (10)	−0.0103 (11)	0.0164 (11)	0.0068 (8)
C3	0.0747 (16)	0.0684 (15)	0.0581 (12)	−0.0179 (12)	0.0285 (12)	−0.0009 (10)
C10	0.0397 (10)	0.0547 (11)	0.0572 (11)	0.0100 (8)	−0.0044 (8)	−0.0157 (9)

### Geometric parameters (Å, º)

**Table:**

S1—C13	1.6744 (17)	C5—C11	1.472 (3)
N1—C12	1.283 (2)	C8—C9	1.412 (2)
N1—N2	1.3528 (19)	C8—H8	0.9300
C6—C1	1.394 (2)	C9—C10	1.367 (3)
C6—C5	1.405 (2)	C9—H9	0.9300
C6—C7	1.410 (2)	C14—H14A	0.9600
O1—C11	1.213 (2)	C14—H14B	0.9600
N2—C13	1.362 (2)	C14—H14C	0.9600
N2—H2	0.8600	C1—C2	1.409 (3)
C7—C8	1.363 (2)	C1—C10	1.411 (3)
C7—C12	1.461 (2)	C2—C3	1.367 (3)
C13—N3	1.312 (2)	C2—H2A	0.9300
N3—C14	1.449 (2)	C4—C3	1.403 (3)
N3—H3	0.8600	C4—H4	0.9300
C12—C11	1.515 (2)	C3—H3A	0.9300
C5—C4	1.364 (3)	C10—H10	0.9300
			
C12—N1—N2	116.93 (13)	C7—C8—H8	120.9
C1—C6—C5	123.08 (16)	C9—C8—H8	120.9
C1—C6—C7	123.95 (16)	C10—C9—C8	122.95 (18)
C5—C6—C7	112.96 (15)	C10—C9—H9	118.5
N1—N2—C13	120.78 (14)	C8—C9—H9	118.5
N1—N2—H2	119.6	N3—C14—H14A	109.5
C13—N2—H2	119.6	N3—C14—H14B	109.5
C8—C7—C6	118.78 (16)	H14A—C14—H14B	109.5
C8—C7—C12	134.48 (16)	N3—C14—H14C	109.5
C6—C7—C12	106.73 (13)	H14A—C14—H14C	109.5
N3—C13—N2	116.67 (15)	H14B—C14—H14C	109.5
N3—C13—S1	125.49 (13)	C6—C1—C2	115.65 (19)
N2—C13—S1	117.84 (13)	C6—C1—C10	115.92 (16)
C13—N3—C14	124.05 (16)	C2—C1—C10	128.43 (18)
C13—N3—H3	118.0	C3—C2—C1	121.1 (2)
C14—N3—H3	118.0	C3—C2—H2A	119.4
N1—C12—C7	125.20 (14)	C1—C2—H2A	119.4
N1—C12—C11	127.56 (15)	C5—C4—C3	117.9 (2)
C7—C12—C11	107.22 (14)	C5—C4—H4	121.1
C4—C5—C6	119.92 (18)	C3—C4—H4	121.1
C4—C5—C11	132.73 (18)	C2—C3—C4	122.4 (2)
C6—C5—C11	107.34 (15)	C2—C3—H3A	118.8
O1—C11—C5	129.05 (16)	C4—C3—H3A	118.8
O1—C11—C12	125.21 (16)	C9—C10—C1	120.19 (17)
C5—C11—C12	105.74 (14)	C9—C10—H10	119.9
C7—C8—C9	118.19 (17)	C1—C10—H10	119.9
			
C12—N1—N2—C13	178.14 (14)	C6—C5—C11—C12	−0.95 (18)
C1—C6—C7—C8	−1.1 (2)	N1—C12—C11—O1	−0.7 (3)
C5—C6—C7—C8	−179.95 (15)	C7—C12—C11—O1	−179.08 (17)
C1—C6—C7—C12	178.56 (15)	N1—C12—C11—C5	179.18 (16)
C5—C6—C7—C12	−0.34 (19)	C7—C12—C11—C5	0.76 (18)
N1—N2—C13—N3	2.3 (2)	C6—C7—C8—C9	1.7 (2)
N1—N2—C13—S1	−178.08 (12)	C12—C7—C8—C9	−177.74 (17)
N2—C13—N3—C14	−179.01 (17)	C7—C8—C9—C10	−1.3 (3)
S1—C13—N3—C14	1.4 (3)	C5—C6—C1—C2	−1.3 (2)
N2—N1—C12—C7	179.26 (15)	C7—C6—C1—C2	179.96 (16)
N2—N1—C12—C11	1.1 (2)	C5—C6—C1—C10	178.58 (16)
C8—C7—C12—N1	0.8 (3)	C7—C6—C1—C10	−0.2 (2)
C6—C7—C12—N1	−178.75 (15)	C6—C1—C2—C3	0.6 (3)
C8—C7—C12—C11	179.24 (18)	C10—C1—C2—C3	−179.2 (2)
C6—C7—C12—C11	−0.29 (17)	C6—C5—C4—C3	−0.7 (3)
C1—C6—C5—C4	1.4 (3)	C11—C5—C4—C3	178.57 (19)
C7—C6—C5—C4	−179.72 (17)	C1—C2—C3—C4	0.0 (4)
C1—C6—C5—C11	−178.08 (15)	C5—C4—C3—C2	0.1 (3)
C7—C6—C5—C11	0.83 (19)	C8—C9—C10—C1	0.0 (3)
C4—C5—C11—O1	−0.5 (4)	C6—C1—C10—C9	0.7 (2)
C6—C5—C11—O1	178.88 (19)	C2—C1—C10—C9	−179.5 (2)
C4—C5—C11—C12	179.7 (2)		

### Hydrogen-bond geometry (Å, º)

Cg is the centroid of ring C1/C6–C10.

**Table:**

*D*—H···*A*	*D*—H	H···*A*	*D*···*A*	*D*—H···*A*
N2—H2···O1	0.86	2.03	2.7178 (19)	136
N3—H3···N1	0.86	2.26	2.6437 (19)	107
N3—H3···S1^i^	0.86	2.64	3.4407 (15)	156
C4—H4···O1^ii^	0.93	2.47	3.246 (2)	141
C2—H2*A*···*Cg*^iii^	0.93	2.76	3.502 (2)	137

Symmetry codes: (i) −*x*+1, *y*−1/2, −*z*+1/2; (ii) *x*+1/2, −*y*+1/2, −*z*; (iii) *x*+1/2, −*y*−1/2, −*z*.
